# Editorial: Regulation and Manipulation of Nutrient-Controlling Genes in Crops

**DOI:** 10.3389/fgene.2020.00164

**Published:** 2020-02-27

**Authors:** Shuijin Hua, Maximiller Dal-Bianco, Zhong-Hua Chen

**Affiliations:** ^1^Institute of Crops and Nuclear Technology Utilization, Zhejiang Academy of Agricultural Sciences, Hangzhou, China; ^2^Laboratório de Bioquímica Genética de Plantas, 212, BIOAGRO and Departamento de Bioquímica e Biologia Molecular, Universidade Federal de Viçosa, Vicosa, Brazil; ^3^School of Science and Health, Western Sydney University, Penrith, NSW, Australia; ^4^Hawkesbury Institute for the Environment, Western Sydney University, Penrith, NSW, Australia

**Keywords:** crop, nutrition, gene, regulation, manipulation

Agriculture, food, and health sectors are largely disconnected because little has been done to consider the complex relationship between agricultural production, food consumption, human nutrition, health, and the food supply chain. As society develops, a healthy diet has becoming increasingly important for humans. Unhealthy diets and nutritional imbalance are highly correlated with several common chronic diseases, such as obesity, cardiovascular disease, and some cancers (Lee et al., 2011). The staples of a healthy diet usually come from crops, and the nutritional qualities of these crops, including the chemical composition of food crops, are essential considerations for a healthy diet. For example, the ratio between amylose and amylopectin in rice and the ratio between saturated and unsaturated fatty acids—particularly the percentage of oleic acid—are both related to human health (Goddard et al., 1984; Mozaffarian et al., 2006). Another major issue is the presence, or absence, of some nutritional components that are essential for human health, such as lysine in rice and tocopherol in the oils (Wang and Galili, 2016; Martin and Li, 2017). All of these require a better understanding of genetic regulation and the interaction between those genes encoding these traits (Martin and Li, 2017).

This Research Topic focuses on the genetic control of important nutrients in crops, the identification of important QTLs combining large effective modern genetic tools such as GWAS analysis, multiple omics analysis on nutrients, gene-controlling nutrients function assays, and the manipulation of genes, such as gene transferring and genome editing. The aim of this Research Topic is to identify key genes and understand the genetic control of nutrients in order to provide healthy foods for humans. The Research Topic includes three original research articles, one systematic review, and one mini review on the subject of carbohydrate-containing and oil-bearing crops.

Fang et al. reviewed the genetic control of malting quality in barley. Malting quality is a very complex quantitative trait, which is composed of many indicators, such as malt extract and diastatic power. Till now, more than 60 quantitative trait loci (QTLs) had been identified. The identification of loci is particularly useful for marker-assisted selection to improve the malting quality. Because there were many interactions between those indicators, as a result, the selection of proper loci or genes controlling malting quality is essential for the combination of beneficial loci and removal of negative effect loci. Barley malting quality can be improved by genetic manipulation with great feasibility because most of the loci are distributed on the seventh chromosome, suggesting the trait may have a chromosome hot spot.

Besides genetic control, crop quality is increasingly affected by environmental contamination from heavy metals and the like. Recently, some functional genes responsible for the heavy metal uptake, transaction, and re-distribution have been identified, such as the rice *OsLsi1* and *OsARM1*, which were summarized by Deng et al.. Due to the fact that the functions of the respective gene in each step of the heavy metal absorption, transportation, and allocation—as well as the eventual movement into the grain in cereal crops—were elucidated, it has become easier for researcher to select candidate genes for interrupting cadmium and arsenic accumulation in grains. Overexpression of these candidate genes under specific or constitutive promoters has confirmed their functions in cadmium and arsenic accumulation. Although genetically modified crops are the concern of many people, there are alternative ways of using mutational methods to create desirable materials with ideal traits. The other way to enhance this possibility is to identify more genes that controlling heavy metal accumulation in harvested tissues for human foods.

Complex crop quality traits hinder research progress in the thorough elucidation of the regulation network; however, this also provides opportunities to identify more genes regulating crop quality traits. One of the challenges for gene identification is to establish more low-cost and efficient identification technologies. Zheng et al. have developed a method to identify a locus with a complex trait, such as leaf shape in pea, using a combination of specific locus amplified fragment sequencing (SLAF) and bulked segregant analysis (BSA). Another advantage of the method is the detection of QTLs without a reference genome. Using this method, two QTLs responsible for leaf morphology were mapped on linkage group 7. Thus, efficient detection methods are great tools to identify quality related genes or molecular markers for marker-assisted breeding.

Apart from the SLAF-BSA method to identify QTLs, Zhu et al.'s work showed the genetic diversity of olive via genotyping-by-sequencing technology. The method was used to analyze the genetic diversity of olive with 57 germplasms from different growth regions mainly in the central Mediterranean. Because of their close relationship among five varieties in China, it is important to utilize the germplasms from other countries as parental lines in breeding programs to improve olive oil quality in the future. Olive is widely accepted by consumers due to its high nutritional value with a high percentage of oleic acid in seed fatty acid. However, the use of cheaper alternatives, such as rapeseed, for high oleic acid content in crops is on the rise (Ishaq et al., 2017). Long et al. have revealed the mutation of *FAD2* resulted in the exceptionally high oleic acid content, which was increased up from 63 to 85% in *Brassica napus*. Two single-nucleotide polymorphisms (SNPs) in *BnFAD2-1* and *BnFAD2-2* were identified to be responsible for the blocking of conversion from oleic acid to linoleic acid. Therefore, the utilization of mutations with high oleic acid content rapeseed germplasm will be an alternative economical application for future food consumption both for its high nutritional quality and yield.

In summary, it is very well-known that agriculture will face big challenges in the next decades to supply food for an increasing population while at the same time not harming the environment. Therefore, it is important to understand the complex pathways and regulatory networks involved in nutrient-controlling genes in crops to be able to manipulate and produce an improved cultivar. The present Research Topic has demonstrated a collection of new findings in the field showing the importance of improving the quality of the genetic material to delivery beneficial food to attend the requirements to a healthy diet.

## Author Contributions

This manuscript was written by SH with contributions from MD-B and Z-HC.

### Conflict of Interest

The authors declare that the research was conducted in the absence of any commercial or financial relationships that could be construed as a potential conflict of interest.
